# Improvements in episodic future thinking methodology: Establishing a standardized episodic thinking control

**DOI:** 10.1371/journal.pone.0214397

**Published:** 2019-03-28

**Authors:** Kelseanna Hollis-Hansen, Sara E. O’Donnell, Jennifer S. Seidman, Spencer J. Brande, Leonard H. Epstein

**Affiliations:** 1 Department of Pediatrics, University at Buffalo Jacobs School of Medicine and Biomedical Sciences, Buffalo, New York, United States of America; 2 Department of Community Health and Health Behavior, University at Buffalo, Buffalo, New York, United States of America; VU University Medical Center, NETHERLANDS

## Abstract

**Objective:**

Delay discounting (DD) is the choice of a smaller immediate reward over a larger delayed reward, which has been associated with a number of maladaptive behaviors. Episodic future thinking (EFT), the ability to project oneself into the future, is an intervention designed to reduce DD. EFT has reliable effects on DD, but the size of the effect varies, which could be due in part to the use of different control groups. Episodic recent thinking (ERT) serves as a common control for many EFT studies, but the temporal window of “recent” cues ranges from 24 hours ago to 12 days past. Since prior research has shown that retrospection can lead to prospection, it may be important to ensure that EFT controls do not inadvertently lead to prospection for some participants thereby increasing the variability of the control condition. The present study sought to develop a comparison group that standardizes the time frame and experiences that are the basis for the recent thinking control.

**Methods:**

Participants (n = 53, 18–45) were randomized to one of three conditions: EFT, ERT, or standardized episodic thinking (SET). Participants attended a laboratory appointment where they played mobile application games, created cues, and completed a DD task.

**Results:**

There was a significant difference between groups (*p*<0.05), with EFT reducing discounting more than either control (p<0.05), and no differences between ERT and SET (p>0.05).

**Conclusion:**

This study established that SET provides an alternative control in EFT studies and provides the advantage of standardizing the participant’s recent experience without changing the relationship between EFT and recent thinking controls.

## Introduction

A large body of literature has shown that episodic future thinking (EFT) interventions modify delay discounting (DD) [1–3], the preference for smaller immediate rewards over the preference for larger delayed rewards [4]. Steep discounting of the future has been associated with many unhealthy behaviors, such as cigarette smoking [5, 6], problematic drinking [7, 8], drug use [9], and obesity [10]. It has also been associated with risky behaviors that are believed to pose a threat to public health, such as neglecting sunscreen, regular dental exams, and using one’s seatbelt [11]. Thus, if we can shift a person’s temporal orientation to be more future-oriented, this could translate to a reduction in maladaptive impulsive behaviors and an increase in health-promoting behaviors.

EFT involves the creation of vivid and positive cues that can expand the temporal window such that as people make decisions in a DD task they are more likely to choose the larger distal reward. In addition to changes in DD, EFT has also been associated with changes in one’s demand for cigarettes, food, and alcohol [3, 12, 13]. EFT has also improved clinical outcomes, for example, reduced eating in the laboratory [14, 15] and in a food court [16] have been observed.

As noted by Schacter and colleagues [17, 18] during EFT people engage in four adaptive behaviors including far-sighted decision-making, emotional regulation (in particular, reducing anxiety), improved prospective memory, and greater spatial navigation. Unhealthy behaviors often have positive immediate consequences (e.g. enjoyment of eating, high from cocaine), but lead to serious distal outcomes (e.g. morbidity, mortality). Therefore, the ability to execute these adaptive behaviors, in particular, the ability to make decisions that benefit future outcomes could promote healthy behavior change.

One of the most commonly used control groups for EFT is episodic recent thinking (ERT) where participants vividly describe events in their recent past in the same way participants generate EFT cues. This condition controls for episodic thought and the procedures used to construct episodic cues, highlighting the temporal differences between EFT and ERT (19). However, consistent and comparable control groups have not always been used across EFT studies and among the studies which employ the ERT control, the retrospective time periods used when generating cues vary widely from three time points in the past 24-hours to three time points within the past 12 days. It is currently understood that the brain accesses past semantic and episodic memories and collates those memories to develop a vivid image of one’s own personal future [17–19]. While the brain is constantly coming up with new ideas and projections, they are believed to be devolved from a collection and reorganization of past experiences. While this phenomenon would be useful in real-world decision-making, it may introduce prospection into usual ERT study conditions if those ERT conditions include cues that go too far back in time. It’s unclear at exactly what point in time retrospection promotes prospection as well as what other internal and external factors may trigger past memories to influence prospective thought.

To illustrate the variability of controls used in EFT studies, we present a brief review of publications that examined whether EFT manipulations can reduce DD. This mini-review was informed by a recent systematic review [2]. We followed similar search and review procedures as those described by the authors [2], except that we used the terms “Episodic Future Thinking” “EFT” Episodic thinking” “Prospective thinking” in conjunction with the DD terms they mention. We identified 19 studies, 10 of which were in the review [2] and added 9 additional studies. We calculated Cohen’s *d* effect sizes and corrected for small sample size bias using Hedge’s *g*. This review showed that only three studies used the same control group, a recent control condition that had participants generate cues over the past 24-hours [3, 12, 19]. The rest of the studies used present events (e.g. over the next 24 hours) [20], no control cues [21–25], cues generated based off of standardized reading material [14, 26] or cues about what money could buy [27]. There were also cues about meals eaten in the recent past [15] and recent/past events that ranged in time from 60 hours [28] to 12 days ago [29] and different time periods in-between [13, 30–33]. Effect sizes varied from Hedge’s *g* = 0.26 [26] to 1.40 [14] **(Table 1 provides more details)**. If we examine just the three studies with cues focused on the participant’s past 24-hours, effect sizes still varied from a medium effect (e.g. 0.49) [19] to a large effect (e.g. 0.85) [12], which highlights that using an ERT control may introduce unnecessary variability into EFT studies. The pooled standard deviation is the denominator in Cohen’s *d* effect size calculations, and greater variability in the control condition (e.g. larger standard deviations) could produce smaller effect sizes.

**Table 1:** Control groups in prior episodic future thinking (EFT) studies.

Study	Manipulation	Control	DV	Effect Size^a^
Benoit et al. (2011)	Imagine spending money in the future	Imagine what money could buy	%LLR	0.31
Daniel et al. (2013a)	Imagine future events	Imagine scenes from Pinocchio	AUC	0.26^b^
Daniel et al. (2013b)	Imagine future events	Imagine scenes from a travel blog	AUC	1.40^b^
Daniel et al. (2016)	Imagine future events	Imagine recent events (past 24 hours)	PMD	0.49
Dassen et al. (2016)	Imagine future events involving food	Imagine recent events involving food/ unconstrained events	*k*	0.48^b^
Kwan et al. (2015)	Imagine future events	No cues	AUC	-
Lin & Epstein (2014)	Imagine or neutral future events	Imagine present events (over the next 24 hours)	*k*	0.45
Liu et al. (2013)	Imagine simulated future events	No cues	%SSR	0.63
O’Donnell, Daniel, and Epstein (2017)	Imagine future events with or without a goal (two groups)	Imagine recent events with or without a goal (past 84 hours, two groups)	AUC	0.81^b^
O’Donnell et al. (2018)	Imagine future events with and without a process (two groups)	Imagine recent events with and without a process (past 86 hours, two groups)	AUC	1.25^b^
O’Donnell, Hollis-Hansen, & Epstein (2018)	Imagine future events (two groups, cues matched or unmatched to DD time periods)	Imagine recent events (past 60 hours, two groups, cues matched or unmatched to usually presented DD time periods)	AUC	0.46^b^
Palombo et al. (2015)	Imagine spending reward in the future	No event cues	LLR	0.75
Peters & Buchel (2010)	Episodic cues present	No cues	*k*	-
Rung & Madden (2018^a^)	Imagine one future event within the following year	Imagine a recent event that occurred between 12 PM and 4 PM yesterday	IP 6-month	0.33
			IP 1-year	0.67
Sasse, Peters, Buchel, & Brassen (2015)	Imagine meeting person in the future	No event cues	*k*	0.26^b^
Snider, LaConte, Bickel (2016)	Imagine future events	Imagine recent events (past 24 hours)	AUC	0.85
Stein et. al (2016)	Imagine future events	Imagine recent events (past 24 hours)	AUC	0.65
Stein et al. (2017)	Imagine future events (one cue 7–12 months in the future vs. three cues at 1, 2–6 months, and 7–12 months, two groups)	Imagine recent events (one cue 7–12 days ago vs. three cues 1, 2–6 days, and 7–12 days ago, two groups)	ln *k*	0.46^b^
Stein, Tegge, Turner, & Bickel (2017)	Imagine future events	Imagine recent events (past 12 days)	ln *k*	-
Sze et al. (20107)^c^	Imagine future events	Imagine recent events (past 6 days)	ln *k*	0.93

DV = Dependent variable, IP = Indifference points at 6-months and 1-year, %LLR = % Larger later reward, ln*k* = log *k* value, PMD = Proportion of max delay, %SSR = % Smaller sooner reward,

^a^ Effect sizes were calculated using Hedge’s *g* to account for smaller sample sizes for some studies,

^b^ Effect sizes averaged across experiments or similar conditions (e.g. if 2 x 2 factorial, averaged across EFT groups),

^c^ We included data from Experiment 1 only, as Experiment 2 used other narrative manipulations (e.g. negative income shock),— = sufficient data was not provided to calculate an effect size

In an attempt to standardize the methods used in the study of EFT and to reduce the potential for the control procedure to evoke prospection, we tested a novel standardized episodic thinking (SET) group in a study of online grocery purchasing behavior [34]. We were concerned that the vivid imagining of one’s recent past (e.g. one week ago) could trigger episodic thoughts about the future thus motivating control participants to buy more healthful food as well. If recalling a meal eaten the weekend prior, it could theoretically lead to EFT about one’s future meals, thus the participant could be more restrictive and healthful in their purchasing during a grocery shopping task. The novel SET control retained the same process of generating cues that include a personalized recent experience, but standardizes the content of that recent experience (e.g. standardizes the episodic thinking across participants) and focuses it on an experience unrelated to food that occurred within the past 15 minutes.

However, we wanted to further pilot the SET control and compare it to a typical ERT group. In the present study participants in the SET control group played three of their top-rated mobile application games for five minutes each and generated cues which asked them to vividly describe the experience of playing the games in the laboratory. To equate the recent experience across both groups, the EFT and general ERT groups also played the same mobile application games, but the EFT group simulated positive future events for their cues and the ERT group focused on positive recent events. All of the groups recited their respective cues before completing a $100 adjusting-amount DD task [35] and were asked to keep their cues in mind while they were making decisions. We hypothesized that the EFT group would discount the future less than the ERT and SET groups and anticipated that the two control groups would have similar rates of discounting thereby establishing the validity of using the new SET control in future EFT intervention studies.

## Methods

### Participants

We conducted an a priori power analysis using an effect size from a study that compared two EFT groups to two ERT control groups [30] with area under the curve (AUC) [4, 36] as the dependent variable. The O’Donnell et. al study resulted in a Cohen’s *d* effect size of 0.89, and from this we calculated that the present three group study needed 17 subjects per group or 51 participants to produce a similar EFT effect size with an alpha of 0.05 and 0.80 power. We anticipated the different control procedures to result in similar levels of discounting as our goal was not to show differences between the control groups, but rather that a standardized control was equivalent to a recent thinking control that has been used in many studies.

Potential participants were recruited through unpaid internet advertisements (e.g. Craigslist, Facebook), community fliers (e.g. open retail, university, and community college message boards) and emails to members of the Division of Behavioral Medicine participant database. The recruitment materials directed potential participants to an eligibility screening, which was hosted on SurveyMonkey.com^®^. Participants were selected as follows:

Participants were excluded if they were younger than 18 or older than 45, as age has been shown to influence delay discounting [11]. A brief demographic questionnaire, that had participant’s self-report their birth date, gender, and race/ethnicity was included in the eligibility screener to collect this information.

Potential participants were presented with pictures and titles of the six mobile application games that they would be asked to play during the study session (e.g. Super Mario, Flow Free, Bubble Witch, Geometry Dash, Solitaire, and Trivia Crack). Participants rated each game on a 5-point Likert scale that gauged their liking of the games and willingness to play the games. Those who rated 3 or more games below a 3 for liking or willingness were excluded and those who rated a 3 or above were included, as we did not want one’s liking of the game to influence the effectiveness of the intervention or lead to a confounded control.

In the present study we wanted to test the EFT effect in a population that exhibited greater discounting of the future to ensure that the EFT intervention works for people who are steep discounters. Therefore, a one-item delay discounting measure asked participants whether they would rather have $50 now or $75 in three months, similar to the one-item delay discounting measure used by Reimers et. al, which was associated with a number of risky behaviors [11]. We excluded participants who selected $75 in three months and included participants who selected $50 now.

Individuals with untreated psychopathology, depression and anxiety were excluded because these illnesses have been associated with a difficulty in developing positive future-oriented cues [37–38] and reduced specificity when generating future events [39]. One two-part self-report item was used to gauge participant’s mental health status, “Do you currently have any psychological conditions (such as a psychological disorder, anxiety, depression, or ADHD)? If yes, please describe (when diagnosed and treatments)”. Those who reported no mental health issues and/or treated and stable conditions were included.

Potential participants who had previously participated in a study with EFT cue generation within the past six months were excluded to ensure there were no carryover effects from a prior study. One self-report item asked participants if they had recently participated in a study involving the generation of sentence cues.

Those who were eligible were contacted by their preferred method of contact and scheduled for a study appointment.

Participants (N = 53) were non-smokers, aged 18–45. See **Table 2**for detailed participant characteristics.

**Table 2:** Participant characteristics.

	EFT	SET	ERT	p
	n = 18	n = 17	n = 18	
*Age (Mean ± SD*, *years)*	29.0 ± 8.4	27.4 ± 6.6	31.9 ± 10.3	.30
*Income (Mean ± SD*, *dollars)*	59167 ± 53340	53824 ± 29513	73944 ± 57311	.45
*SSS (Mean ± SD)*	5.7 ± 1.4	5.5 ± 1.5	5.4 ± 1.5	.76
*Education (Mean ± SD*, *years)*	14.7 ± 2.2	14.3 ± 2.1	14.8 ± 2.1	.74
*Race (n*, *%)*				.40
White	12 (66.67%)	9 (52.94%)	8 (44.44%)	
Non-white	6 (33.33%)	8 (47.06%)	10 (55.56%)	
*Ethnicity (n*, *%)*				.37
*Hispanic*	1 (5.56%)	0 (0.00%)	0 (0.00%)	
Non-Hispanic	17 (94.44%)	17 (100.00%)	18 (100.00%)	.
*Sex (n*, *%)*				.62
Female	13 (72.22%)	10 (58.82%)	13 (72.22%)	
Male	5 (27.28%)	7 (41.18%)	5 (27.78%)	
*Mobile Game Liking (Mean ± SD)*	3.6 ± 0.4	3.6 ± 0.7	3.6 ± 0.5	.95
*Imagery measures (Mean ± SD)*				
Frequency	3.6 ± 0.9	3.3 ± 1.0	3.3 ± 0.9	.41
Vividness	3.4 ± 0.9	3.2 ± 1.2	3.3 ± 0.9	.77

SSS = Subjective social status

### Procedures

This study was approved by the Social and Behavioral Institutional Review Board of the University at Buffalo. Consent of participants was obtained in a private room and participation occurred from February to August of 2018.

After completing the consent process, participants were randomly assigned to one of three groups: an episodic future thinking group (EFT), an episodic recent thinking group (ERT), and a standardized episodic thinking group (SET). Participants then played 6 mobile application games for 1 minute each and rated their liking of each game on a 5-point Likert scale, where 1 was “do not like at all” and 5 was “like very much”.

The six games available were Super Mario, Flow Free, Bubble Witch, Geometry Dash, Solitaire, and Trivia Crack. Super Mario is similar to the well-known Nintendo console game, and is considered a “platformer” game, or a game where a player has to move their character between suspended platforms while avoiding barriers. In Super Mario the player is moving a 3-dimensional Mario through “Super Mario World”, collecting tokens and averting obstacles that cause the player to lose the game. Flow Free is a puzzle game which has the player connect similarly colored dots while ensuring not to break any of the other connections with the goal of completing the puzzle. Bubble Witch is a bubble shooting puzzle game where participants guide a witch’s wand to shoot a colored bubble at a group of similarly colored bubbles with the objective of removing the bubbles and getting to the top. Geometry Dash is also a platformer game, but the game is faster paced than Super Mario and the “character” is a square with the objective of getting to the end of the level without crashing into any other obstacles (e.g. other geometrical shapes). Solitaire is an electronic version of the popular card game (also known as “Patience”) in which players sort cards in alternating color order and then stack them by suit. Trivia Crack is a single-, two- or multi-player trivia game where participants spin a wheel that randomly selects a trivia category. Participants then have 20 seconds to answer a question related to entertainment, geography, science, sports, history or art. After rating all six games, participants chose their three favorite mobile games and played each of those three games for an additional 5 minutes. Once they finished playing the third game, participants created cues with a study staff member.

The participants’ cues varied depending on their group assignment. The EFT group created positive and personalized episodic future simulation cues in two steps. Participants were first asked to identify a positive event that they were looking forward to and could vividly imagine that could really happen or that they had planned for one month, six months, and one year from that day (e.g. a wedding, birthday party, family vacation, outdoor adventure, etc.). Participants were then directed to rate and describe the event. The events were rated on a 5-point Likert scale for both liking and vividness of imagined event. Any participants rating below a 3 were asked to simulate a new event. Participants then described their future event in as much detail as possible in order to help them vividly simulate their future while making choices. The sample **EFT cue** that was given to participants reads as follows:

“In about 1 month I am at the park with my friend Alex. We are at Letchworth State Park, sitting by the waterfront. I am admiring the beautiful scenery. I am feeling relaxed and enjoying the nice weather outside.”

The ERT group created episodic recent thinking cues in the same two steps as the EFT group, by identifying a recent positive event and rating that event on their liking and vividness. ERT participants were directed to create cues for recent past events that happened 24 hours (1 day), 72 hours (3 days), and 144 hours (6 days) ago. The sample **ERT cue** reads as follows:

“About 72 hours ago I was at the park with my friend Alex. We were at Letchworth State Park, sitting by the waterfront eating lunch. I was admiring the beautiful scenery. I was feeling relaxed and enjoyed the nice weather outside.”

The SET participants created cues about the three mobile application games that they had selected and played for 5 minutes each at the beginning of their study appointment. A sample **SET cue** is as follows:

“About 5 minutes ago I was playing Bubble Witch in a beige study room at UB. I was playing as a witch and guiding her wand towards similarly colored bubbles in order to pop them. I was releasing the owls as I popped the bubbles. I was feeling excited as I was making my way to the top.”

Once finished creating cues, participants in all groups completed an adjusting amount DD task. The task asked people to choose between a smaller variable reward now and a larger fixed reward in the future ($100) at six different delay periods: one day, one week, one month, six months, one year, and two years [30]. Each time period had six trials of monetary choices. The amount available in the present varied by how participant’s responded to the question prior, for example, the first trial is always $50 now or $100 at the later time period, and then the second trial will either decrease the value of the present reward by 50% if the participant selected the $50 or increase the present value by 50% if the participant selected the $100. The point at which the participant was indifferent to the smaller immediate reward and larger delayed reward (e.g. valued them the same) was the indifference point for each time period, and those points were used to plot a curve and our major dependent variable was the area-under-the-curve because of the ease in interpretation [35]. A lower AUC (closer to 0) indicates greater future discounting while a higher AUC (closer to 1) indicates greater future consideration [4].

The participants were asked to read their cues before beginning the DD task and reminded to keep their cues in mind while they made their decisions in the task. At the end of each time period, participants were asked to rate the frequency and vividness of their thoughts about their cues during the task. This was done on a 5-point Likert scale, asking “How often did you think about your cues during the task?” and “How vivid were your thoughts about your cues?” where one was “not at all” for frequency and “not vivid” for vividness and five was “very often” for frequency and “very vivid” for vividness. If the participant rated either vividness or frequency below a three, they were asked to read their cues again before returning to the task.

### Other measures

Upon completion of the DD task, participants were directed to a separate computer to complete a computer based survey. The survey included the following measures:

#### Demographic variables

Age, income, education, sex, race, occupation, and marital status of the participants were obtained through the use of the sociodemographic questionnaire from the MacArthur Network [40].

#### Subjective Social Status (SSS)

A two-item questionnaire was used to assess subjective social status, which asks participants to view a photo of a ladder with the number 10 at the bottom and the number one at the top. Participants are instructed to define their community in the way that makes sense to them. They are then asked to think of the bottom of the ladder “10” as the people who have the lowest standing in their community and those at the top of the ladder “1” as the people with the highest standing in their community. They are then asked to place themselves on the ladder where they think they stand relative to other people in their community at the present moment. They are given virtually identical instructions for question two, but they are instructed to think of the ladder as representing where people stand in the entire United States. We included SSS as another measure of SES [40].

### Analytic plan

Between groups analysis of variance (ANOVA) was calculated for all continuous variables and Chi-square tests were computed for categorical variables to assess whether groups differed on any important characteristics. None of the variables differed by group and therefore no covariates were included in the final model. In order to test our first hypothesis, whether EFT improves DD, we conducted a between groups ANOVA with group assignment as the independent variable and AUC as the dependent variable. In order to test our second hypothesis, whether there would be differences between the two control groups, follow-up linear contrasts were used to determine specific group differences in AUC. To test group differences between individual indifference points (IP) we conducted a mixed ANOVA with group as the between factor and IP (e.g. 1 week, 1 month…) as a within factor. Post hoc linear contrasts were used to test between group differences and whether groups differed at each time point. Weber and Popova’s independent-samples equivalence procedure was used to test whether the control groups were equivalent [41]. Laken’s [42] has suggested the best effect size boundary to consider is the smallest effect size of interest. In our data, a 0.10 difference in AUC (e.g. a 0.30 AUC versus a 0.40 AUC) results in a Cohen’s *d* of 0.50. Therefore, we used a Cohen’s *d* of 0.50 (Δ = .50) in our independent-samples equivalence procedures.

## Results

No significant differences were observed in how much participants liked playing the mobile application games (*F*(2, 50) = 0.06, *p* = 0.95), how much participants thought about their cues during the DD task (*F*(2, 50) = 0.92, *p* = 0.45), or how vivid participant’s thoughts were about the events described in their cues during the DD task (*F*(2, 50) = 0.26, *p* = 0.77). There were no differences among other important participant characteristics that would justify including additional variables in the analysis.

**Fig 1:**
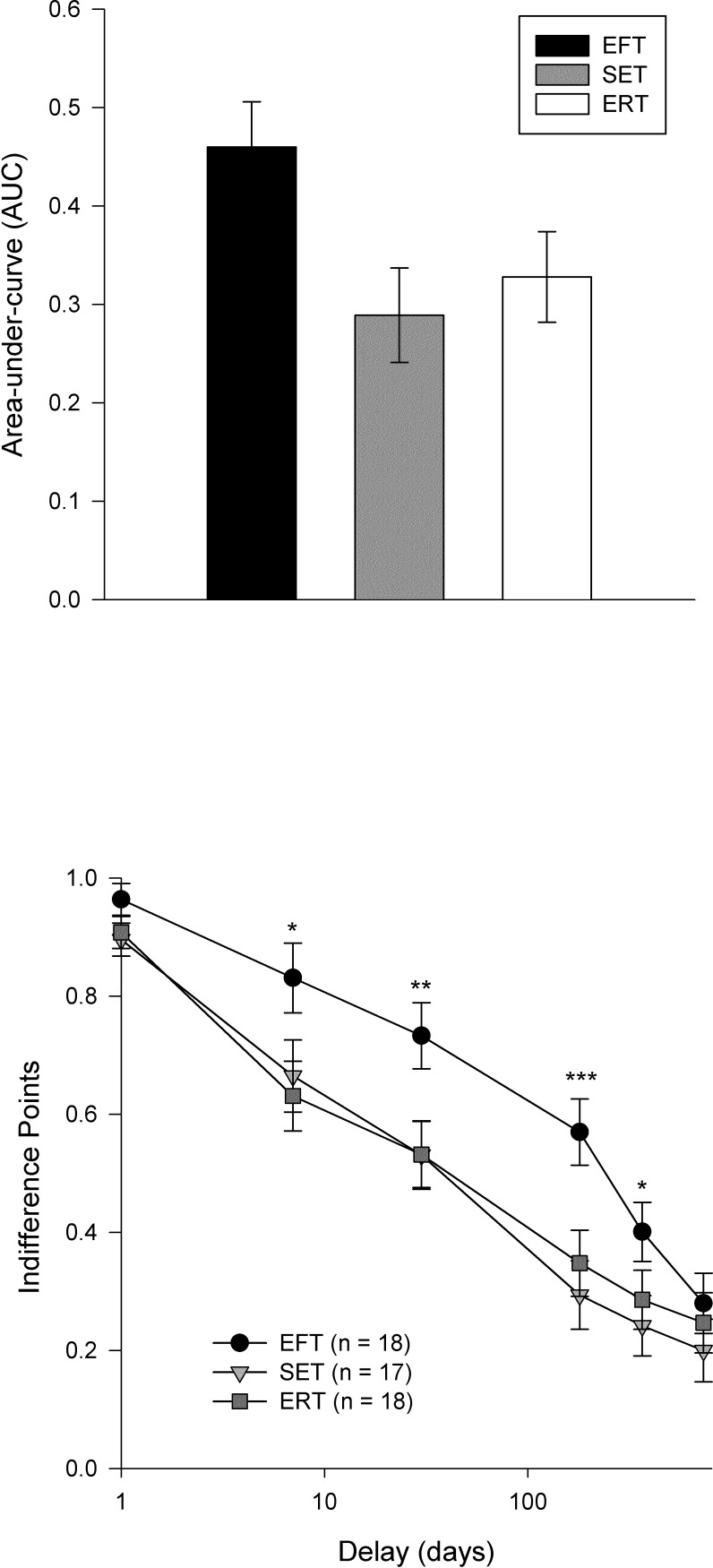
Delay discounting area-under-the-curve (mean ± SE) by randomized group assignment. Participants randomized to the EFT group discounted the future less than those who were randomized to either control group (*p* < 0.05). The two groups did not differ (p > 0.05). Indifference points (mean ± SE) by randomized group assignment for each of the time delays (e.g. one day, one week, one month, six months, one year, and two years). * p ≤ .05 ** p ≤ .01 *** p ≤.001.

Significant differences in AUC were observed, mean AUC’s were: EFT .46 ± .21, ERT .33 ± .23, and SET .29 ± .13 ([mean ± SD]), *F*(2, 50) = 3.67, *p* < 0.05 (**Fig 1**). Contrasts showed that the EFT group made more far-sighted decisions than SET (*F*(1, 50) *=* 6.61, *p* < 0.05, η_p_^2^ = 0.12, Hedge’s *g* = 0.99), and ERT (*F*(1, 50) *=* 4.05, *p* < 0.05, η_p_^2^ = 0.08, Hedge’s *g* = 0.68), while the two ERT groups did not differ from one another *F*(1, 50) = 0.34, *p* > 0.05, η_p_^2^ = 0.007, Hedge’s *g* = 0.21). The independent-samples equivalence procedure showed that the SET and ERT groups are equivalent (*t*(33) = 0.60, Δ = .50, *p* = 0.02).

There was one statistical outlier in the general ERT group (an AUC of .99, which indicates no future discounting). When this person was removed the overall ANOVA showed a larger EFT effect (*F*(2, 49) = 5.74, *p* < 0.01; η_p_^2^ = 0.19), no difference between the two control groups (*F*(1, 49) = 0.00, *p* > 0.05, η_p_^2^ = 0.000, Hedge’s *g* = 0.00), and that the two control groups were equivalent (*t*(32) = 0.00, Δ = .50, *p* = 0.005).

The mixed ANOVA of the indifference points showed overall main effects for group (*F(2*, *50)* = 4.55, *p* < 0.05) and time (*F(5*, *50)* = 166.19, *p* < 0.001). In addition, there was an interaction of Group x Time (*F(10*, *250)* = *p* < 0.05) suggesting that there may be differential effects at different temporal intervals. As shown in Fig 1, there were no significant differences between the ERT group and the SET group at any of the indifference points (*p*’s *>* 0.05).

A post-hoc contrast looking at EFT versus both control groups showed that EFT effects appear to be most pronounced for the one week (*F*(*1*, *50*) = 6.29, *p <* 0.05), one month (*F*(*1*, *50*) = 8.40, *p <* 0.01), six month (*F*(*1*, *50*) = 13.14, *p* = 0.001), and one year (*F(1*, *50)* = 5.02, *p* < 0.05) time periods (**Fig 1**). There is no difference at the one day (*F(1*, *50)* = 3.45, *p* = 0.07) or two-year intervals (*F(1*, *50)* = 0.79, *p* = 0.38). However, effects for the one week and one-year indifference point differed slightly by control group.

There were one week differences for the SET control (*F*(*1*, *50*) = 3.81, *p =* 0.057) and the ERT control (*F*(*1*, *50*) = 5.69, *p <* 0.05), one month differences for the SET control (*F*(*1*, *50*) = 6.22, *p <* 0.05) and ERT control (*F*(*1*, *50*) = 6.32, *p <* 0.05), six months differences for the SET control (*F*(*1*, *50*) = 11.89, *p =* 0.001) and ERT control (*F*(*1*, *50*) = 7.90, *p <* 0.01), and one year differences for the SET control only (*F*(*1*, *50*) = 4.99, *p <* 0.05), not the ERT control (*F*(*1*, *50*) = 2.67, *p >* 0.05).

## Discussion

The present study replicated the finding that EFT helps participants make more far-sighted decisions in a laboratory DD task among an impulsive sample of the population. Additionally, we confirmed that the SET control is a valid control for future EFT intervention studies, as SET reliably differs from EFT, but is not significantly different than ERT. An advantage of the SET control is that it standardizes the recent experience participants recall during the DD task and reduces the possibility that participants could engage prospection to the same effect that usual ERT participants might as they recall experiences that occurred a week or two in the past. In addition, SET would provide the same recent experience for all participants across studies, which increases the power of meta-analytic studies that would compare EFT to common controls. We found that there were similar ratings in how frequently participants thought about their cues and how vivid their thoughts were about their cues regardless of group assignment, further confirming the validity of using the SET group as an EFT intervention control.

Analyses of the indifference points suggest that cues do not need to be matched to the time delay to produce an effect, given that there were significant differences at the one-week and six-month time points, which are not cued during the EFT manipulation. This result replicates a recent finding that EFT cues do not need to match the discounting time delays in order to improve DD [28]. We found significant differences between the EFT group and the control groups at the one week, one month, six months, and one year delays. There were no significant differences between the ERT and SET groups at any of the time periods.

EFT consistently shows a positive and significant effect on DD outcomes, but the effects vary across studies [2]. This may be due in part to differences in the implementation of EFT, but in a comparison of EFT studies with the same EFT methods, the effect sizes would still vary based on differences in the control groups. Standardization of control conditions may be important for future meta-analyses of EFT, so that different interventions can be compared to a common control. While ERT can be considered a common control group, as we have shown the temporal characteristics of ERT conditions can vary. In addition, even if everyone is asked to recall a pleasant experience that occurred over the past 24 hours, that experience will be very different across participants. SET instead standardizes the recent experience of all participants. In addition, it may be important to consider how ERT would be used in clinical populations with the goal of changing a health behavior. In clinical studies there may be a clearer relationship between what the participant has recently experienced (e.g. eating at a party, drinking alcohol during a night out with friends) and the behaviors they are trying to change.

It is important to consider limitations to the interpretation of the results. The sample size was relatively small, and the study was powered to show differences between EFT and the two control groups, not between the control groups. The differences between the two groups was small, with a Cohen’s *d* effect size of 0.21, which would require 788 participants, or 394/group to show significant between group differences. Second, we used mobile games as the format for standardizing the participant’s recent experience. In the Supermarket Study (34) and in the present sample this was perceived as a highly enjoyable activity, but it is possible that this may not be a universally enjoyable activity, which could compromise the effectiveness of SET or limit the sample of participants.

The SET may be a better control than a general ERT group because it standardizes and manufactures a similar recent experience for all participants thereby reducing the variability in the recent experience. The new control replicates that it is episodic **future** simulation that is changing decision-making by controlling for all of the other aspects of cue generation that could be attributed to change: the control provides an experience that is enjoyable to all participants and follows a similar cue generation and recitation process as the intervention group. The control also provides an episodic recent simulation that is able to be frequently and vividly imagined that is unrelated to the outcome of interest. The SET would be a useful control for researcher’s seeking to study the influence of EFT on DD as well as other health behaviors and outcomes, such as alcohol intake, cigarette smoking, eating behaviors, and grocery shopping.
